# A review of sleep deprivation studies evaluating the brain transcriptome

**DOI:** 10.1186/2193-1801-3-728

**Published:** 2014-12-11

**Authors:** Alisa S Elliott, Jason D Huber, James P O’Callaghan, Charles L Rosen, Diane B Miller

**Affiliations:** School of Medicine, West Virginia University, Morgantown, WV USA; School of Pharmacy, West Virginia University, Morgantown, WV USA; Toxicology and Molecular Biology Branch, CDC-NIOSH, 1095 Willowdale Rd, Morgantown, WV 26505 USA

**Keywords:** Sleep deprivation, Transcriptome, Sleep disruption, Rodent

## Abstract

Epidemiological studies show a positive association between adequate sleep and good health. Further, disrupted sleep may increase the risk for CNS diseases, such as stroke and Alzheimer’s disease. However, there has been limited progress in determining how sleep is linked to brain health or how sleep disruption may increase susceptibility to brain insult and disease. Animal studies can aid in understanding these links. In reviewing the animal literature related to the effects of sleep disruption on the brain, we found most of the work was directed toward investigating and characterizing the role of various brain areas or structures in initiating and regulating sleep. In contrast, limited effort has been directed towards understanding how sleep disruption alters the brain’s health or susceptibility to insult. We also note many current studies have determined the changes in the brain following compromised sleep by examining, for example, the brain transcriptome or to a more limited extent the proteome. However, these studies have utilized almost exclusively total sleep deprivation (e.g., 24 out of 24 hours) paradigms or single short periods of limited acute sleep deprivation (e.g., 3 out of 24 hours). While such strategies are beneficial in understanding how sleep is controlled, they may not have much translational value for determining links between sleep and brain health or for determining how sleep disruption may increase brain susceptibility to insult. Surprisingly, few studies have determined how the duration and recurrence of sleep deprivation influence the effects seen after sleep deprivation. Our aim in this review was to identify relevant rodent studies from 1980 through 2012 and analyze those that use varying durations of sleep deprivation or restriction in their effort to evaluate the effects of sleep deprivation on the brain transcriptome and to a more limited extent the proteome. We examined how differences in the duration of sleep deprivation affect gene and protein expression to better understand the full consequences of repeated sleep disruption on the brain. Future research needs to consider and emphasize how the type and extent of the sleep deprivation exposure impacts the conclusions reached concerning the influence of sleep disruption on the brain.

We identified relevant studies between 1980 and 2012 by searching the electronic databases of PubMed, Medline (Ovid), Embase (Ovid), and Web of Science using the terms “sleep” AND “disrupt”, “deprivation”, “restrict”, “fragment”, “loss”, “disturb”, “disorder”, “dysfunction”, “brain”, “cortex”, striatum”, hypothalamus”, “hippocampus”, “gene”, “protein”, “genomics”, “proteomics”, “polymerase chain reaction”, “pcr”, “microarray”, “molecular”, “rodent” “rat”, “rats”, “mouse”, “mice”. All searches were limited to rodent studies in English and the reference lists of retrieved articles were searched for additional pertinent studies.

**Electronic supplementary material:**

The online version of this article (doi:10.1186/2193-1801-3-728) contains supplementary material, which is available to authorized users.

## Introduction

Sleep, one of the most conserved behaviors, consumes approximately one third of a person’s life, yet the purpose of sleep is still not fully understood. In an effort to understand the function of sleep, researchers predominately study the effects of sleep deprivation (SD) and sleep restriction (SR) in humans and animals. Initially, increased sleepiness was thought to be the main consequence of sleep loss and its presence accounted for the various effects of sleep loss such as cognitive impairment. Consequently, researchers did not focus on the possible short and long-term pathophysiological consequences of sleep loss that could impact overall health (Kushida 2006). Much of the early emphasis was on neurobehavioral recovery following sleep loss and ways to treat and hasten recovery (Kushida 2006; Lamond et al. 2007). More recently, epidemiological studies have suggested that a person’s overall health is influenced by sleep patterns throughout life, with a positive association seen between shortened sleep duration and morbidity and mortality (Qureshi et al. 1997; Ayas et al. 2003; Ferrie et al. 2007; Ikehara et al. 2009; Luyster et al. 2012). People who report habitually short sleep durations, defined as sleep durations less than six hours each night, have an increased prevalence of type 2 diabetes, hypertension, obesity, cardiovascular disease, and stroke (Qureshi et al. 1997; Ayas et al. 2003; Schultes et al. 2005; Spiegel et al. 2005; Gottlieb et al. 2006; Ferrie et al. 2007; Cappuccio et al. 2008; Chen et al. 2008; Ikehara et al. 2009; Kim and Jo 2010; Sabanayagam and Shankar 2010; Luyster et al. 2012; Chaput et al. 2007). Further, a study in Finland found that workers engaged in shift work (SW), that is, work outside regular daytime hours, had a higher incidence of stroke suggesting some aspect of shift work increases the vulnerability of the brain (Nurminen and Karjalainen 2001). Disrupted sleep is often a byproduct of SW resulting in shift workers being more likely to have shorter sleep durations than people who work regular daytime hours (Luckhaupt et al. 2010). According to the 2010 National Health Interview Survey (NHIS), over 40 million employed U.S. adults report habitually short sleep durations. Furthermore, the American Academy of Sleep Medicine and the Sleep Research Society define this the prolonged state of shortened or mistimed sleep as *chronic sleep deficiency* and recently reported their concern for its increase in the population and the probable negative health outcomes as a result (Luyster et al. 2012). Despite the increasing research emphasis on understanding sleep unfortunately the function of sleep remains elusive. Although, adequate sleep appears to be necessary for good health the relationship(s) between sleep and health remain unclear and difficult to objectively measure.

Cognitive impairment due to sustained wakefulness or SR has been the focus of much research in the sleep research arena. Many studies, including dose–response studies have evaluated the cumulative build-up of cognitive performance deficits with extended SD or SR (Van Dongen et al. 2003). Despite the advances in determining the cognitive consequences of insufficient sleep the physiological function of sleep is still not well characterized or understood. Advancement over the last 20 years in “omics” methods, such as transcriptomics utilizing real-time polymerase chain reaction (PCR) and cDNA microarrays, as well as proteomics to a lesser extent has aided in a better understanding of the differences between sleep, waking, and sleep deprived states at the molecular level. Many studies demonstrate differences in the molecular characteristics of brain following various forms of sleep disruption. Microarrays, specifically, have become widely used in sleep research to evaluate and compare transcriptomic profiles of sleep-wake and sleep deprived states with an initial emphasis on characterizing the sleep and awake states. Together, these studies have yielded thousands of candidate genes involved in sleep homeostasis and/or function (Terao et al. 2003a; Terao et al. 2003b; Cirelli et al. 2006; Terao et al. 2006; Mackiewicz et al. 2007; Maret et al. 2007; Kilduff et al. 2008; Mackiewicz et al. 2009; Thompson et al. 2010; Veasey 2010; Datta et al. 2011; Mongrain et al. 2011). Many of these genes are now considered sleep-state regulated and belong to classes of genes involved in specific cellular functions, such as synaptic maintenance and plasticity (Taishi et al. 2001; Nelson et al. 2004; Maret et al. 2007; Das et al. 2008; Mallick and Singh 2011; Tadavarty et al. 2011; Franco-Perez et al. 2012; Singh et al. 2012; Volkow et al. 2012), metabolism (Basheer et al. 2001; Kong et al. 2002; Mackiewicz et al. 2003; Nikonova et al. 2010; Petit et al. 2010; Everson and Szabo 2011; Martins et al. 2011; Barf et al. 2012), stress response (Meerlo et al. 2002; Terao et al. 2003b; Sgoifo et al. 2006; Brown and Naidoo 2010; Kalinchuk et al. 2010), and neuroprotection (Weil et al. 2009; Mongrain et al. 2010; Dattilo et al. 2011; Wisor et al. 2011). From this research, several theories of the physiological function of sleep have developed that have the potential to begin to explain the link between sleep habit and overall health. It is important to note, however, that many of these studies only looked at the molecular consequences of the sleep state after one episode of short-term sleep deprivation. How the duration and frequency of sleep deprivation influences the outcome has received little attention as factors important to consider, especially when analyzing and interpreting the results. Evaluating the data from the studies in a duration-dependent construct will provide insight into how the brain reacts to sleep deprivation as it cumulates. Therefore, this review aims to analyze the existing literature on sleep deprivation’s effect on the transcriptome by using duration of sleep deprivation as the chief independent variable. Although the review concentrates on the impact of sleep deprivation on the brain transcriptome, proteomic studies are included when warranted. To begin the data sources and study selection criteria are described and followed by a brief background on methods used to identify and assess sleep.

### Data sources and study selection

An extensive search of the electronic databases of PubMed, Medline, Embase, and Web of Science was conducted to identify animal studies (1980–2012) that assessed the effects of SD on gene and protein expression in the brain. The terms “sleep” AND “disrupt”, “deprivation”, “restrict”, “fragment”, “loss”, “disturb”, “disorder”, “dysfunction”, “brain”, “cortex”, striatum”, hypothalamus”, “hippocampus”, “gene”, “protein”, “genomics”, “proteomics”, “polymerase chain reaction”, “pcr”, “microarray”, “molecular”, “rodent” “rat”, “rats”, “mouse”, “mice” were used in the searches. The reference lists of included studies were also searched for pertinent papers. The first and last author determined the inclusion/exclusion criteria. The animal studies were limited to rat and mouse studies for easier generalizability across studies. All methods utilized to cause SD were included. However, studies modeling sleep disorders, such as sleep apnea and insomnia, were excluded. Furthermore, gene expression needed to be determined for multiple, not single, genes in order to provide a more complete profile of the SD transcriptome. Any paper investigating the impact of sleep on a brain region was accepted, but cortex became the focal point since it was the region of interest in the majority of studies. Publication dates for the searches ranged from 1980 to July 2012, and all searches were restricted to English.

### Identifying and assessing sleep

Sleep is widely defined as a rapidly reversible state of greatly reduced responsiveness and immobility (Siegel 2005; Allada and Siegel 2008). The advent of the electroencephalogram (EEG) signify the beginning of modern sleep research. By placing electrodes on the scalp, the EEG records the voltage changes produced by ionic currents within the thousands of neurons in the cerebral cortex. The voltage changes are recorded continuously, resulting in data in the form of a wave. Wave profiles, determined by the wave’s amplitude and frequency, reflect neuronal activity and correspond with levels of consciousness, including stages, or depth, of sleep. EEG recordings can be used in mammals as a way to identify sleep and its various stages.

More recently, a set of distinct behaviors has become accepted for defining sleep given the difficulty of transplanting electrodes to record EEGs in many species. One behavior required is reduced motor activity and is usually accompanied by a species-specific sleep posture. A second recognized sleep behavior is a reduced or total lack of response to stimuli during sleep that regularly evokes a response when awake. Thirdly, sufficient stimuli should cause the rapid reversal from sleep to wake states. The ability to quickly reverse back to the wake state is important as it differentiates sleep from coma. Finally, a balance between sleep and wake states, termed *sleep homeostasis*, needs to be apparent. The existence of sleep homeostasis is supported by the presence of *sleep* propensity and *recovery* sleep. Sleep propensity, also termed sleepiness, is the likelihood of an individual to fall asleep. Sleep propensity increases as wakefulness continues. Recovery sleep is the longer and deeper sleep that occurs after SD. A species exhibiting all these behaviors are considered to have sleep-like states. By including behavior criteria to identify sleep, researchers have been able to identify several other species that engage in sleep-like states, such as the fruit fly *Drosophila melanogaster* (Hendricks et al. 2000; Shaw et al. 2000), zebra fish *Danio rerio* (Zhdanova et al. 2001; Yokogawa et al. 2007), and roundworm *Caenorhabditis elegans* (Van Buskirk and Sternberg 2007; Raizen et al. 2008).

Sleep function and homeostasis are primarily investigated through SD studies where various techniques are implemented in an attempt to prevent the animal from sleeping (Table 1). For instance, some SD techniques place the rodent on a constantly rotating drum or platform (“disk-over-water” or DOW) where the animal must continuously walk to avoid water and cannot sleep (Rechtschaffen et al. 1999; Coenen and van Luijtelaar 1985). Less physically demanding methods place the rodent inside an automated running wheel or on a platform (“moving platform”) that is activated at sleep onset via EEG monitoring (Fenzl et al. 2007). The gentle handling method, where the rodent is lightly prodded or brushed when sleep is observed, is considered to be one of the least stressful methods and reduces the amount of physical activity required of the rodent (Fenzl et al. 2007). Unfortunately, this method is not automated and can be taxing on the researcher when implemented for long periods of time. Consequently, gentle handling is usually only used for short term (i.e., acute) SD studies of 8 hours or less. Each technique introduces its own set of extraneous variables into the study and the reader should know that although these methods can produce a substantial amount of sleep deprivation none of these result in total SD. Even the EEG monitoring methods have some “lag-time” between the recognition of a sleep state and waking the animal. Further, sleep processes such as microsleeps, local sleep, increased hippocampal spike rates and increases in the EEG recording amplitude can intrude during the deprivation period even though the procedures are successful in keeping animals awake for a large period of time (Friedman et al. 1979; Vyazovskiy et al. 2011). For example, EEG monitoring of rats deprived of sleep by the gentle-handling method indicate they are kept awake ~ 94% of the deprivation period (Leenaars et al. 2011). Thus, the technique used to produce a protracted awake state needs to be carefully considered in evaluating the impact of sleep deprivation on various outcomes. It is certain that the choice of a sleep deprivation method ultimately influences the classes of genes responsible for specific cellular functions (e.g., plasticity, etc.) due to the differences in activity, stress, etc. associated with a given deprivation technique. Investigators in choosing a deprivation procedure must balance the benefit(s) of the technique (i.e. increased animal processing for automated techniques) with the cost (e.g., an increased stress response) of its use.

**Table 1:** **Studies evaluating the consequences of sleep deprivation on the brain**

Reference	SD method	Conditions	Species; sex	Age	Time of sacrifice*	Expression method	Brain area
Naidoo N. *et al*., 2005. (Naidoo et al. 2005)	Gentle handling	3 h, 6 h, 9 h and 12 h SD - beginning at lights-on	C57/B6 mouse; ♂	10 weeks	Immediately after SD	WB	CTX
Maret S. *et al*., 2007. (Maret et al. 2007)	Gentle handling	6 h SD - beginning at multiple time points	C57BL/6 J; AKR/J; DBA/2 J Mouse; ♂	12-13 weeks	During last 30 min of SD	MA, QPCR	Whole Brain
Conti B. et al., 2007. (Conti et al. 2007)	Gentle handling	24 hours SD	Sprague–Dawley rats; ♂	Adult (250-300 g)	Immediately after SD	MA	PFC, FCTX, AMY, HYPO, HIPP, DRN, LC
Thompson C. et al., 2010. (Thompson et al. 2010)	Gentle handling	6 h SD; 4 h RS after 6 h SD - beginning at lights-on	C57BL/6 J mouse; ♂	9-11 week	Immediately after SD or RS	MA, ISH	ORB, SCN, HCRT, TMN, PMCo, ENT, LC
Cirelli C. and Tononi G., 1999. (Cirelli and Tononi 1999b)		3 h SD; 3 h spontaneously asleep (S); 3 h spontaneously awake (W)	Wistar Kyoto (WKY) rats; ♂			MA	CTX
Cirelli C. et al., 2004. (Cirelli et al. 2004)	Gentle handling	8 h SD - beginning at lights-on; spontaneously asleep (S); spontaneously awake (W)	WKY rats; ♂		Immediately after SD, 6 am for W rats	Microarray (pooled samples); real-time qPCR (pooled samples)	CTX
Cirelli C et al., 2006. (Cirelli et al. 2006)	Gentle handling for s-SD; DOW for l-SD	8 h SD (s-SD) - beginning at lights-on; 7d SD (l-SD)	WKY rats; ♂	Adult (300-450 g)	Immediately after SD	MA, QPCR	CTX
Mackiewicz M., et al., 2007. (Mackiewicz et al. 2007)	Gentle handling	3 h, 6 h, 9 h and 12 h SD - beginning at lights-on	C57BL/6 J mice; ♂	10-12 weeks	Immediately after SD	MA	CTX, HYPO
Nikonova E., et al., 2010. (Nikonova et al. 2010)	Gentle handling	h3 and 12 h SD - beginning at lights-on	C57BL/6 J mice; ♂	8-10 weeks	Immediately after SD	WB, QPCR	CTX
Terao A., et al., 2003. (Terao et al. 2003a)	Gentle handling	6 h SD; 4 h RS after 6 h SD - beginning at lights-on	C57BL/6 J mice; ♂	10-12 weeks	Immediately after SD or RS	WB, QPCR	BF, TH, HYPO, CTX, CB, P, MD
Cirelli C. and Tononi G., 2000. (Cirelli and Tononi 2000a)	Gentle handling	1-9 h SD – beginning at lights-on	WKY rats; ♂	Adult (300 g)	Immediately after SD	DD, RPA	Right CTX, Right HIPP
Cirelli C. and Tononi G., 2000. (Cirelli and Tononi 2000b)	Gentle handling	8 h SD; spontaneously asleep (S); spontaneously awake (W)	WKY rats; ♂	Adult (300-350 g)	Immediately after SD; end of light period for S; end of dark period for W	DD, RPA	CTX
Taishi P. et al., 2001. (Taishi et al. 2001)	Gentle handling	8 h SD; 2 h RS after 8 h SD	Spague-Dawley rats; ♂	Adult (320-350 g)	Immediately after SD or RS	RT-PCR, QPCR	CTX, HIPP
Terao A., et al., 2006. (Terao et al. 2006)	Gentle handling	6 h SD; 2 h RS after 6 h SD - beginning at light onset	Wistar rats ♂	2-3 months	Immediately after SD or RS	MA	CTX, BF, HYPO
Cirelle C. and Tononi G., 2004. (Cirelli and Tononi 2004)	Gentle handling for short-term SD; DOW for long-term SD	8 h SD (s-SD); 7d ( l-SD) - beginning at lights-on	WKY rats; ♂	Adult (300-450 g)	Immediately after SD	RPA, QPCR	Right CTX (also liver and muscle)
Cirelle C. and Tononi G., 1998. (Cirelli and Tononi 1998)	Gentle handling	3 h SD; 3 h spontaneously asleep (S); 3 h spontaneously awake (W)	WKY rats; ♂	Adult (300-350 g)	Immediately after SD; During dark phase for W rats	DD, RPA	Left CTX
Mackiewicz M., et al., 2003. (Mackiewicz et al. 2003)	Gentle handing	12 h SD beginning at lights-on (7 am)	Fischer rats ♂	2 months (180-200 g)	Multiple time points throughout day – all with time-matched controls	WB	CTX, LC, DRN, TMN, VDB, HDB, VLPO

*Most of the studies had multiple times of sacrifice; therefore specific times were not listed. Instead, time of sacrifice in relation to when SD occurred is provided. All studies did include time-matched controls.

### Established hypotheses

The regulation of the sleep-wake cycle has been one of the central questions of sleep research. The presence of sleep or sleep-like states across phyla suggests that sleep is a behavior essential to survival. Additionally, sleep deprivation can cause death sooner than food deprivation in both rats (Rechtschaffen et al. 1983) and *Drosophila* (Shaw et al. 2002), further supporting sleep’s necessity. As such, sleep must be regulated and maintained. Unfortunately, the precise mechanisms underlying sleep behavior are still not fully elucidated.

The most established and accepted model explaining the alteration between sleep and wake is the two-process model of sleep regulation (Borbely 1982; Borbely and Achermann 1999). Presented in the early 1980’s, this model posits that two distinct processes control the alteration: the circadian clock and the sleep homeostat. Circadian mechanisms, termed Process C, involve sustained rhythmic oscillations for sleep propensity across a 24 hour period. Process C is based in the suprachiasmatic nucleus (SCN) and has been well characterized. Circadian clock genes are the main modulators of Process C and regulate sleep tendency to occur at ecologically appropriate times. Additionally, clock genes have been shown to modulate other behaviors, such as feeding, seeming to play a role in regulating basic survival behaviors (McGlincy et al. 2012). Interestingly, sleep deprivation and limited sleep influences the expression of the clock genes themselves (Wisor et al. 2002; Franken et al. 2006; Moller-Levet et al. 2013).

The homeostatic mechanisms, termed process S, regulate sleep propensity across the sleep-wake cycle, in that the drive to sleep increases as wakefulness continues. Accordingly, sleep propensity decreases at sleep onset. Studies using EEG have shown an increase in delta power after longer periods of wakefulness, providing support for the regulatory role of Process S (Dijk and Beersma 1989; Franken et al. 2001; Dijk and Lockley 2002) in the sleep-wake cycle. One important distinction between these two processes is that the sleep-wake ratio drives Process S mechanisms, whereas Process C is self-sustaining, independent of the sleep-wake ratio. It should be noted that chronic sleep restriction initially instigates enhanced sleep and slow wave activity during the opportunity for sleep but these effects of sleep restriction habituate rapidly (Kim et al. 2013).

Process S is also called the sleep homeostat because it regulates the level of sleep propensity. The mechanisms involved in Process S are less characterized but suggest a restorative and/or repairing function of sleep. Thus, the two-process model of sleep regulation suggests that Process S is responsible for the function of sleep and Process C is responsible for influencing the timing of sleep. A number of hypotheses on the function of sleep have been proposed based on this model of dualistic control.

### Energy hypothesis

The function of sleep, according to Benington and Heller’s energy hypothesis, is to restore the brain energy stores that are depleted during wakefulness (Benington and Heller 1995). Wakefulness is associated with increased neuronal activity and consequently, increased energy demands. These demands are thought to result in depletion of energy stores during wakefulness. One important energy source for neuronal activity is ATP, which is required for neuronal depolarization. ATP is produced in the inner mitochondrial membrane by the oxidative phosphorylation system (OXPHOS). Mitochondrial (Nadh2 and Cox1) and nuclear (Cox4 and Atp5a) genes encoding proteins involved in OXPHOS, such as *Nadh2*, are upregulated after only 3 hours of sleep deprivation (Cirelli and Tononi 1999a; Terao et al. 2003a; Nikonova et al. 2010) (Table 2, Additional file 1: Table S1). These observations support the theory that wakefulness increases the demand for energy.

**Table 2:** **Genes shown in the literature to be upregulated in the cortex after sleep deprivation**

	3 hours	6 hours	8 hours	12 hours	24 hours	7 days total SD
**Immediate early genes/transcription factors**	*Arc* (Cirelli and Tononi 2000a; Cirelli 2002) *Fosb* (Cirelli and Tononi 1998; Cirelli 2002) *Egr1* (Cirelli and Tononi 1998; Cirelli 2002) *Homer1a* (Mackiewicz et al. 2007; Maret et al. 2007)	*Arc* (Terao et al. 2006; Thompson et al. 2010; Maret et al. 2007) *Fosb* (Maret et al. 2007; Terao et al. 2003a) *Fra-2* (Terao et al. 2003a; Terao et al. 2006; Maret et al. 2007) *Junb* (Terao et al. 2003a) *Egr1* (Terao et al. 2003a; Terao et al. 2006) *Egr2* (Maret et al. 2007) *Egr3* (Terao et al. 2003a; Terao et al. 2006; Maret et al. 2007) *Homer1a* (Maret et al. 2007; Mackiewicz et al. 2007)	*Arc* (Taishi et al. 2001; Cirelli 2002; Cirelli and Tononi 2000a, [b]; Cirelli et al. 2006) *Fosb* (Cirelli et al. 2006) *Fra* (Cirelli et al. 2006) *CHOP* (Cirelli and Tononi 2000b; Cirelli 2002; Cirelli et al. 2004) *IER5* (Cirelli and Tononi 2000b; Cirelli 2002) *Egr1* (Cirelli and Tononi 2000b; Cirelli 2002; Cirelli et al. 2006) *Ngf1-b* (Cirelli and Tononi 2000b; Cirelli 2002; Cirelli et al. 2006) *Egr2* (Cirelli et al. 2006) *N-ras* (Cirelli 2002; Cirelli and Tononi 2000b) *Stat3* (Cirelli and Tononi 2000b; Cirelli 2002) *Homer1a* (Cirelli et al. 2006)	*Homer1a* (Mackiewicz et al. 2007)	*Egr2* (Conti et al. 2007) *Homer1a* (Conti et al. 2007)	*Arc* (Cirelli et al. 2006) *Fosb* (Cirelli et al. 2006) *Fra* (Cirelli et al. 2006) *Egr1* (Cirelli et al. 2006) *Ngf1-b* (Cirelli et al. 2006) *Egr2* (Cirelli et al. 2006) *Homer1a* (Cirelli et al. 2006) *Ania-1* (Cirelli et al. 2006)
**Energy metabolism/energy balance**	*Cox1* (Cirelli 2002; Cirelli and Tononi 1998, 1999a, [b], 2000b; Cirelli et al. 2004; Cirelli and Tononi 2004; Naidoo et al. 2005) *Cox4* (Cirelli et al. 2004; Nikonova et al. 2010) *Atp5a* (Cirelli et al. 2004; Cirelli and Tononi 2004) *Nadh2* (Cirelli 2002; Cirelli and Tononi 1998, 1999a, [b])	*Nrf-1* (Nikonova et al. 2010)	*Glut1* (Cirelli 2002; Cirelli and Tononi 2000b) *Vgf* (Cirelli 2002; Cirelli and Tononi 2000b; Cirelli et al. 2006) *Ucp2* (Cirelli et al. 2004; Cirelli and Tononi 2004)	*Cox1* (Nikonova et al. 2010) *Cox4* (Nikonova et al. 2010) *Atp5a* (Nikonova et al. 2010) *Ucp2* (Nikonova et al. 2010)		*Vgf* (Cirelli et al. 2006)
**Chaperones/heat shock proteins/stress response**		*BiP* (Maret et al. 2007; Terao et al. 2006; Mackiewicz et al. 2007; Naidoo et al. 2005) *Erp72* (Terao et al. 2006) *Grp94* (Maret et al. 2007; Terao et al. 2006) *Hsp90ab1* (Maret et al. 2007) *Hspb1* (Maret et al. 2007) *Gadd45a* (Mackiewicz et al. 2007) *Gadd45b* (Maret et al. 2007) *Calr* (Mackiewicz et al. 2007; Maret et al. 2007) *Dnajc3* (Mackiewicz et al. 2007) *Dnajb5* (Mackiewicz et al. 2007) *Dnajb11* (Mackiewicz et al. 2007) *Dnajc1* (Mackiewicz et al. 2007) *Hsp105* (Mackiewicz et al. 2007) *Hspa1a* (Mackiewicz et al. 2007) *Hspa1b* (Mackiewicz et al. 2007) *Nrf2* (Nikonova et al. 2010)	*BiP* (Cirelli and Tononi 2000b; Cirelli 2002; Cirelli et al. 2004; Naidoo et al. 2005) *PERK* (Cirelli et al. 2004) *Erp72* (Cirelli and Tononi 2000b; Cirelli 2002) *Hspa9* (Cirelli and Tononi 2000b; Cirelli 2002) *Grp94* (Cirelli and Tononi 2000b) *Hsp60* (Cirelli and Tononi 2000b; Cirelli 2002) *Hsp70* (Cirelli and Tononi 2000b; Cirelli 2002) *Calr* (Cirelli et al. 2006) *Ppp3* (Cirelli et al. 2004)	*BiP* (Naidoo et al. 2005; Mackiewicz et al. 2007)	*Hspb1* (Conti et al. 2007)	*BiP* (Cirelli et al. 2006) *Mrp14* (Cirelli et al. 2006) *Taf9b* (Cirelli et al. 2006) *Cort* (Cirelli et al. 2006) *Hspb1* (Cirelli et al. 2006) *Hspa1a* (Cirelli et al. 2006) *Cryab* (Cirelli et al. 2006) *Gllg15b* (Cirelli et al. 2006) *Mgst1* (Cirelli et al. 2006) *Gpx3* (Cirelli et al. 2006) *CYP4F4* (Cirelli et al. 2006)
**DNA binding/regulation of transcription**		*Dbp* (Maret et al. 2007)				*Dbp* (Cirelli et al. 2006) *Atff5* (Cirelli et al. 2006) *Neurod1* (Cirelli et al. 2006) *c-myc* (Cirelli et al. 2006)
**Vesicle- and synapse-related**			*Scg2* (Cirelli and Tononi 2000b; Cirelli 2002) *Syt4* (Cirelli 2002; Cirelli and Tononi 2000b)			*Scg2* (Cirelli et al. 2006) *VAP1* (Cirelli et al. 2006)
**Growth factors/adhesion molecules**		*Bdnf* (Maret et al. 2007) *Vegfa* (Maret et al. 2007)	*Bdnf* (Cirelli 2002; Taishi et al. 2001; Cirelli and Tononi 2000a, [b]; Cirelli et al. 2006) *TrkB* (Cirelli and Tononi 2000a; Cirelli 2002; Cirelli and Tononi 2000b) *Cntn1* (Cirelli and Tononi 2000b; Cirelli 2002)		*Bdnf* (Conti et al. 2007) *Ifrd1* (Conti et al. 2007)	*Bdnf* (Cirelli et al. 2006) *Ntm* (Cirelli et al. 2006) *Gas* (Cirelli et al. 2006)
**Receptors**			*Adra1a* (Cirelli 2002; Cirelli and Tononi 2000b) *Adrb2* (Cirelli 2002; Cirelli and Tononi 2000b) *Gabrb3* (Cirelli 2002; Cirelli and Tononi 2000b) *Grin2a* (Cirelli 2002; Cirelli and Tononi 2000b) *Glur2* (Cirelli and Tononi 2000b; Cirelli 2002) *Glur3* (Cirelli and Tononi 2000b; Cirelli 2002) *Chrnb2* (Cirelli 2002; Cirelli and Tononi 2000b) *Thrb* (Cirelli and Tononi 2000b; Cirelli 2002) *Itpr3* (Cirelli et al. 2004)			*Ntsr* (Cirelli et al. 2006)
**Enzymes**		*Sgk1* (Maret et al. 2007)	*Sult1a1* (Cirelli 2002; Cirelli and Tononi 2000b) *Mapk81* (Cirelli and Tononi 2000b; Cirelli 2002) *Sgk1* (Cirelli and Tononi 2000b; Cirelli 2002) *Gpd1* (Cirelli et al. 2006) *Fkbpb1a* (Cirelli et al. 2004)		*Sult1a1* (Conti et al. 2007) *Dclk1* (Conti et al. 2007) *Ret* (Conti et al. 2007) *Gpd1* (Conti et al. 2007)	*Sult1a1* (Cirelli et al. 2006) *Alox12* (Cirelli et al. 2006) *Sgk1* (Cirelli et al. 2006) *Fdft1* (Cirelli et al. 2006) *Ptgs2* (Cirelli et al. 2006) *Pdk2* (Cirelli et al. 2006) *Gpd1* (Cirelli et al. 2006) *Nqo1* (Cirelli et al. 2006) *Aldoc* (Cirelli et al. 2006) *Glb* (Cirelli et al. 2006) *Cbs* (Cirelli et al. 2006)
**Hormone/** **Hormone regulation**					*Crhbp* (Conti et al. 2007)	*Crhbp* (Cirelli et al. 2006) *Crh* (Cirelli et al. 2006) *Cort* (Cirelli et al. 2006)
**Other**		*Cdkn1a* (Maret et al. 2007) *Dusp14* (Maret et al. 2007) *Dusp4* (Maret et al. 2007) *Nptx2* (Maret et al. 2007) *Pdia3* (Maret et al. 2007) *Vip* (Maret et al. 2007) *Xbp-1* (Maret et al. 2007)	*tPA* (Taishi et al. 2001) *CaM* (Cirelli 2002; Cirelli and Tononi 2000b) *Ccnd2* (Cirelli and Tononi 2000b; Cirelli 2002) *Lmo-4* (Cirelli and Tononi 2000b; Cirelli 2002) *Mt3* (Cirelli and Tononi 2000b; Cirelli 2002) *Junb* (Cirelli et al. 2006) *Cebpb* (Cirelli et al. 2006)		*Gfap* (Conti et al. 2007) *Anp32a* (Conti et al. 2007) *Mt1a* (Conti et al. 2007)	*tPA* (Cirelli et al. 2006) *IgK* (Cirelli et al. 2006) *Junb* (Cirelli et al. 2006) *Hba1* (Cirelli et al. 2006) *Cebpb* (Cirelli et al. 2006) *Rpl21*(Cirelli et al. 2006) *Alb* (Cirelli et al. 2006) *Npy* (Cirelli et al. 2006) *Hbb* (Cirelli et al. 2006) *Kcnh3* (Cirelli et al. 2006) *Syt12* (Cirelli et al. 2006) *Vip* (Cirelli et al. 2006) *Ptpn1* (Cirelli et al. 2006) *Grifin* (Cirelli et al. 2006) *Ccnd3* (Cirelli et al. 2006) *Klf15* (Cirelli et al. 2006) *Nupl1* (Cirelli et al. 2006) *Ilf3* (Cirelli et al. 2006) *Ptp4a2* (Cirelli et al. 2006) *Ctnnb1* (Cirelli et al. 2006)

Note: This is a compilation from multiple research articles and does not give a complete account of how a particular gene’s expression changes across durations of deprivation. The absence of genes in the 12 and 24 hour columns comes from a gap in the research for those durations and does not imply a decrease in the number of genes upregulated after 12 and 24 hours of sleep deprivation.

Accordingly, energy store depletion should induce sleep. Therefore, it is believed that the increased demand for ATP is initially produced by increasing the activity of OXPHOS. However, as wakefulness continues, ATP stores become depleted and sleep is initiated. Increased ATP expenditure would produce increased levels of adenosine, which has been shown to promote sleep (Porkka-Heiskanen et al. 1997). Furthermore, extracellular levels of adenosine in the cerebral cortex have been shown to increase with sustained wakefulness. Accordingly, adenosine may act as modulator of the sleep-wake cycle and promote sleep when energy levels become depleted. Interestingly, extracellular ATP is believed to also play a role in initiating sleep. Extracellular ATP binds to type 2 purine receptors causing glia to release cytokines (e.g., IL1, TNF) which also act through adenosine to promote sleep (Clinton et al. 2011; Frank 2012).

### Synaptic homeostasis hypothesis

Sleep appears to promote brain plasticity including memory enhancement and stabilization. Although the mechanism for this property of sleep is unknown there are a number of hypotheses for this including the synaptic homeostasis hypothesis or SHY. SHY proposes that sleep regulates synaptic weight and makes the following 4 predictions: 1) synaptic potentiation in several cortical structures is associated with wakefulness; 2) synaptic potentiation has been tied to the regulation of slow-wave sleep; 3) synaptic downscaling is associated with slow-wave activity; 4) synaptic downscaling is tied to the beneficial effects of sleep on neural connections as evidenced by performance (Tononi and Cirelli 2003).

During wakefulness, genes encoding proteins involved in synaptic plasticity, specifically long-term potentiation (LTP), are upregulated during wakefulness and down regulated during sleep. The plasticity-related genes *Arc*, brain-derived neurotropic factor *(BDNF), Homer1a,* and nerve growth factor-induced gene A (*NGFI-A;* also known as *Egr1*) are among the best documented plasticity-related genes to be state-dependent. Specifically, these genes have increased expression during wakefulness and sleep deprivation (Cirelli and Tononi 2000b, [a]; Cirelli et al. 2004). Moreover, a positive correlation was shown in rats between *BDNF* expression and exploratory behavior, even when duration of wakefulness was controlled (Huber et al. 2007). Consequently, synaptic potentiation of cortical networks occurs during wakefulness through BDNF, resulting in a net increase of synaptic strength (Tononi and Cirelli 2003). Although considered state-regulated, *Arc, Homer 1a,* and *NGFI-A* have not been shown to be associated with exploratory behavior.

Additionally, *BDNF* expression increase was associated with increased slow-wave activity (SWA) during subsequent sleep (Huber et al. 2007). According to this hypothesis, synaptic potentiation is also linked to SWA in that synaptic downscaling occurs during this time. Downscaling refers to a proportional reduction in the strength of all synapses onto the same neuron. Therefore, downscaling results in a decrease in synaptic weight without interfering with the relative differences in synaptic strength important for memory traces. Taken together, BDNF counteracts the increase of synaptic strength during waking with a subsequent increase of SWA during sleep, resulting in synaptic downscaling. In this way, BDNF is thought to be a possible modulator of the homeostatic sleep response at the molecular level.

Downscaling of synaptic strength would benefit neuronal function by limiting energy expenditure. Metabolic demands due to neuronal repolarizations following postsynaptic potentials account for approximately 44% of the energy required for the cerebral cortex (Howarth et al. 2012). Increased synaptic strength also increases energy need. Therefore, synaptic downscaling that occurs during sleep maintains energy efficiency of the cerebral cortex. However, there are some concerns about SHY despite experimental evidence supporting this elegant hypothesis. See Frank (Frank 2012) for a comprehensive review of the strengths and weaknesses of the SHY hypothesis.

### Macromolecular biosynthesis hypothesis

In 2007, a microarray study found that the most abundant group of genes upregulated in the cortex and hypothalamus of mice during sleep encoded proteins involved in macromolecule biosynthesis (Mackiewicz et al. 2007). For example, heme biosynthesis in the cortex is one pathway upregulated during sleep. Some of the genes upregulated in this pathway encode enzymes of the heme biosynthesis pathway, proteins that regulate heme level, and heme containing proteins. Structural components of ribosomes, translation initiating factors, and transcripts involved in tRNA activation are among the subcategories of upregulated genes involved in protein synthesis (Mackiewicz et al. 2007). Another biosynthetic pathway upregulated during sleep is protein synthesis. Therefore, sleep seems likely to be the primary state for synthesis of proteins and other macromolecules.

Furthermore, genes encoding enzymes of the cholesterol-synthesis pathway increase progressively during sleep. Specifically, these upregulated genes encode proteins involved in cholesterol uptake and transport, as well as chaperones and transcription factors responsible for regulating transcription of cholesterol-related genes (Mackiewicz et al. 2007). The increase in cholesterol biosynthesis is likely to increase the amount of membrane cholesterol. Cholesterol is a component of the cell membrane and modulates the membrane’s fluidity over the range of physiological temperatures. Furthermore, cholesterol aids in signal transduction through its structural role in membrane microdomians called lipid rafts. Additionally, transcript levels for genes encoding proteins involved in lipid rafts, such as flotilin, are also upregulated during sleep. Lipid rafts modulates signal strength by corralling neurotransmitter receptors with other signaling molecules, increasing the likelihood that they interact (Simons and Toomre 2000). Taken together, sleep is important for membrane stability and signal transduction. These observations suggest that the function of sleep is to repair and replenish in preparation for the upcoming demands of wakefulness.

### Molecular consequences of prolonged sleep deprivation

Acute sleep deprivation up to 6 hours results in gene expression resembling those seen during wakefulness. However, as sleep deprivation is prolonged, gene expression patterns begin to suggest increased cellular stress. Cellular stress can occur when there is an interruption in physiological balances (McEwen 2006), such as those necessary for protein and calcium homeostasis in the ER (Naidoo et al. 2005). Sleep deprivation studies consistently result in increased expression of transcripts associated with stress response in the ER (ie, sleep deprivation can be considered a cellular stressor). The ER is an organelle that regulates protein folding and transport through various pathways and post-translational modifications. Molecular chaperone proteins, such as binding immunoglobulin protein (BiP; also known as GRP78 and HSPA5) and glucose-related protein, 94 kDa (GRP94; also known as HSP90B1), aid in protein folding by stabilizing protein intermediates. Protein misfolding can occur when there is a disturbance in ER homeostasis, causing cellular stress. Accumulation of misfolded or unfolded proteins triggers the UPR.

The UPR is an adaptive mechanism in the ER to control and limit the amount of unfolded proteins that could become toxic and reinstate protein homeostasis. Three main mechanisms make up the UPR. The first involves increasing transcription of chaperone proteins to aid in the proper folding of proteins. Second, protein translation is attenuated by activating the serine-threonine kinase PKR-like ER kinase (PERK), which then phosphorylates the eukaryotic initiation factor 2α (eIF2α). Finally, unfolded proteins are removed from the ER for degradation. If stress is prolonged, these mechanisms cannot restore homeostasis. Apoptotic pathways are initiated by the activation of CHOP (C/EBP homologous protein), JNK (c-jun NH2 terminal kinase), and caspases such as Caspase 9 and ensure elimination of the aberrant proteins.

Sleep deprivation studies consistently show upregulation of genes involved in the UPR (Table 2). Transcript levels of BiP, a key marker of UPR activation, is upregulated in the cerebral cortex after only 6 hours of sleep deprivation in both mice (Naidoo et al. 2005; Mackiewicz et al. 2007) and rats (Cirelli et al. 2004) and continues to stay upregulated after 7 days of total sleep deprivation (Cirelli et al. 2006). Further, PERK expression has also been shown to be upregulated after a short duration of sleep deprivation (Cirelli et al. 2004). However, although the PERK pathway is activated after 6 hours (Naidoo et al. 2005), expression of PERK mRNA increases with 8 hours of sleep deprivation (Table 2). Taken together, these findings lend credence to the theory that sleep is associated with protein translation, with likely attenuation of this during sleep deprivation. Thus, an alteration in protein translation may be a key consequence of sleep deprivation.

## Conclusion

The majority of the studies reviewed were microarray studies which have distinct limitations. Microarrays do not contain all genes in a given genome allowing for the possibility of missing critical genes. Although the newer technologies for transcriptomic analyses (e.g., NextGen) contain the entire genome, both microarrays and NextGen produce large datasets which require bioinformatics approaches to identify relevant pathways. This is illustrated in the paper by Wang (Wang et al. 2010) on sleep deprivation where a variety of data analysis strategies were used to identify pathways impacted by sleep disruption (e.g., synaptogenesis, etc.). While gene expression changes can be identified it is difficult to determine how specific brain cells are impacted by sleep disruption as the tissue evaluated contains a variety of cell types (e.g., neurons, glia, etc.). New approaches such as bacTRAP are beginning to solve these problems (Doyle et al. 2008; Emery and Barres 2008; Heiman et al. 2008).

Sleep research in animals utilizing microarrays and other transcriptomic approaches has begun to yield a better understanding of the influence of the sleep-wake cycle on the brain. Specifically, studies utilizing these modern molecular techniques have shown that the homeostasis or balance between the two states is important for normal brain functioning. When this cycle is disrupted, cellular stress pathways respond to reduce the negative consequences. However, protracted activation of these stress pathways can lead to elimination of these “stressed” cells by apoptosis. Further, the existing studies have efficiently documented the consequences for brain of both short-term and long-term sleep deprivation. However, the existing studies do not allow us to understand the consequences of repeated short-term sleep deprivation – a scenario with more translational impact because it is relevant for the sleep disruption conditions humans are more likely to experience. Although brain is an organ not easily accessed in living humans for transcriptomic or proteomic evaluation there is great interest in examining the blood transcriptome as an accessible window to other organs, including the brain (Moller-Levet et al. 2013; Clinton et al. 2011; Liew et al. 2006; Kohane and Valtchinov 2012). What are the consequences of a repeated activation of these stress pathways? Do they begin activating apoptosis earlier? Or are regular patterns of shorter sleep durations having no specific influence on health? Sleep studies need to begin expanding the limits of sleep deprivation to include more relevant human patterns of sleep limitations and disruptions. Only then can we start locating particular pathways where intervention is possible to modulate the negative effects of chronic sleep deficiency.

## Abbreviations

### Experimental methods

DD: Differential display; ISH: In situ hybridization; MA: Microarray; QPCR: Quantitative PCR; RPA: Rnase protection assay; RT-PCR: Reverse-transcriptase PCR; WB: Western blot.

### Brain regions

AMY: Amygdala; BF: Basal forebrain; CB: Cerebellum; CTX: Cortex; DRN: Dorsal raphe nucleus; ENT: Entorhinal cortex; FCTX: Frontal cortex; HCRT: Hypocretin neurons; HDB: Horizontal limb of the diagonal band of Broca; HIPP: Hippocampus; HYPO: Hypothalamus; LC: Locus coeruleus; MD: Medulla; ORB: Orbital cortex; P: Pons; PFC: Prefrontal cortex; PMCo: Posteromedial cortical amygdala; SCN: Suprachiasmatic nucleus; TH: Thalamus; TMN: Tuberomammillary nucleus; VDB: Verticallimb of the diagonal band of Broca; VLPO: Ventrolateral preoptic area.

## Electronic supplementary material

Additional file 1: Table S1: List of gene symbol, full gene name, and aliases of all genes listed in Table 2. (PDF 106 KB)
