# Supplementary material for: A review of sleep deprivation studies evaluating the brain transcriptome
Source: Springerplus. 2014 Dec 11;3:728. doi: 10.1186/2193-1801-3-728 (PMC4409616; doi:10.1186/2193-1801-3-728)
Supplement: Supplementary file 1 — Additional file 1: Table S1: List of gene symbol, full gene name, and aliases of all genes listed in Table 2. (PDF 106 KB) [file 40064_2014_1531_MOESM1_ESM.pdf]

Title: A Review of Sleep Deprivation Studies Evaluating the Transcriptome
Journal: Cellular and Molecular Life Sciences
Authors: AS Elliott, JD Huber, JP O'Callaghan, CL Rosen, DB Miller
Corresponding Author: Diane B. Miller, CDC-NIOSH, Morgantown, WV dum6@cdc.gov

Gene Symbol	Full Name	Aliases
Adra1a	Adrenergic, alpha-1A receptor	ALPHA1AAR, Alpha-1A adrenoreceptor
Adrb2	Adrenergic beta-2, receptor surface	Beta-2 adrenoreceptor, BETA2AR, catecholamine receptor, B2AR
Alb	albumin	serum albumin
Aldoc	aldolase C, fructose-biphsophate	
Alox12	arachidonate 12-lipoxygenase	
Ania-1	activity and neurotransmitter-induced early gene 1	
Anp32a	acidic nuclear phosphoprotein 32 family, member A	
Arc	activity-regulated cytoskeleton-associated protein	ARC/ARG3.1, KIAA0278
Atf5	activating transcription factor 5	
Atp5a	ATP synthase, complex V	
Bdnf	brain-derived neurotrophic factor	abrineurin, neurotrophin
BiP	Binding immunoglobulin protein	GRp78, Hspa5
Calr	Calreticulin	
CaM	Calmodulin	
Cbs	cystathionine-beta-synthase	HIP4, serine sulfhydrase, beta thionase
Ccnd2	Cyclin D2	G1/S-specific cyclin D2, KIAK0002
Ccnd3	Cyclin D3	D3-type cyclin
Cdkn1a	cyclin-dependent kinase inhibitor 1a	CIP1, CAP20, WAF1
Cebpb	CCAAT/enhancer binding protein (C/EBP), beta	LAP, TCF5, CRP2, IL6DBP
CHOP	DNA-damage-inducible transcript 3	GADD153, C/EBP-homologous protein
Chrnb2	cholinergic receptor, nicotinic, beta 2 (neuronal)	EFNL3, nAChRB2
c-myc	DNA for c-myc, exon 2	
Cntn1	Contactin	F3, contactin 1, neuronal cell surface protein F3, GP135
Cort	Cortistatin	CST-14, CST-17, preprocortistatin
Cox1	Cytochrome C oxidase subunit 1	
Cox4	Cytochrome C oxidase subunit 4	
Crh	corticotropin-releasing hormone	CRF, corticoliberin
Crhbp	corticotropin-releasing hormone binding protein	CRFBP
Cryab	alpha B-crystallin	
Csda	Cold shock domain protein A	DBPA, ZONAB
Ctnnb1	catenin (cadherin-associated protein), beta 1	catenin beta-1, beta catenin
CYP4f4	cytochrome P450 4F4	
Dbp	D site of albumin promoter (albumin D-box) binding protein	DABP, D-site binding protein
Dclk1	doublecortin and CaM kinase-like 1	CL1, CLICK1, serine/threonine-protein kinase DCLK1
Dnajb11	DnaJ (Hsp40) homolog, subfamily B, member 11	EDJ, ERdj3, HEDJ
Dnajb5	DnaJ (Hsp40) homolog, subfamily B, member 5	Hsc40
Dnajc1	DnaJ (Hsp40) homolog, subfamily C, member 1	ERdj1, MTJ1, HTJ1
Dnajc3	DnaJ (Hsp40) homolog, subfamily C, member 3	P58, HP58, PRKRI
Dusp14	dual specificity phosphatase 14	MKP6, MKP-L, mitogen-activated protein kinase phosphatase 6
Dusp4	dual specificity phosphatase 4	MKP-2, MKP2, TYP
Egr1	early growth response 1	NGFI-A, Zif268
Egr2	early growth respons 2	Krox20, AT591
Egr3	early growth respons 3	zinc finger protein pilot
Erp72	Endoplasmic reticulum protein 72	Erp70, Pdia4
Fdft1	squalene synthetase	
Fkbpb1a	FK506 binding protein, 1A 12kDa	PKC12, PPIASE, FKBP1
Fosb	FBJ osteosarcoma viral oncogene B	c-fos, p55, AP-1
Fra	fos-related antigen 1	fra-1, fos-like antigen
Fra-2	fos-related antigen 2	FOSL2, FRA2
Gabrb3	gamma-aminobutyric acid A receptor, beta 3	GABA-alpha receptor beta-2 subunit, ECA5
Gadd45a	growth arrest and DNA-damage-inducible, alpha	DDIT1, GADD45
Gadd45b	growth arrest and DNA-damage-inducible, beta	MYD118
Gas6	growth potentiating factor	
Gfap	glial fibrillary acidic protein	
GIlg15b	global ischemia-induced protein	
Glb	diacetyl/L-xylulose reductase	
Glur2	Glutamate receptor, ionotropic, AMPA 2	GRIA2, glutamate receptor 2, GluR-B
Glur3	Glutamate receptor, ionotropic, AMPA 3	GRIA3, glutamate receptor 3, GluA3
Glut1	Glucose transporter type I	DYT18, GLUT-1DS
Gpd1	glycerol 3-phosphate dehydrogenase 1	GPD-C, GPDH-C
Gpx3	plasma glutathione peroxidase precursor	
Grifin	galectin-related interfiber protein	
Grin2a	glutamate receptor, ionotropic, N-methyl D-aspartate 2A	GluN2A, NMDA receptor subtype 2A
Grp94	Glucose-regulated protein 94	Hsp90B1
Hba1	hemoglobin, α1	
Hbb	Hemoglobin beta	HBD, beta globin chain, beta globin
Homer1a	homer homologue 1	Ves-1, Homer-1
Hsp105	heat shock 105kDa/110kDa protein 1	HSP105A, HSP105B, KIAA0201
Hsp60	Heat-shock protein 60	HLD4, SPG13, GROEL
Hsp70	Heat-shock protein 70	
Hsp90ab1	heat shock protein 90kDa alpha (cytosolic), class B member 1	Hsp90B, Hsp84
Hspa1a	heat shock 70kDa protein 1A	HSP70-1, HSPA1
Hspa1b	heat shock 70kDa protein 1B	HSP70-2, HSP70-1B
Hspa9	heat shock 70kDa protein 9 (mortalin)	Grp75
Hspb1	heat shock 27kDa protein 1	Hsp27
IER5	immediate early response 5	SBBI48
Ifrd1	interferon-related developmental regulator 1	PC4, TIS7
IgK	Ig germline k-chain C-region gene	
Ilf3	interleukin enhancer binding factor 3	
Itpr3	inositol 1,4,5-triphosphate receptor, type 3	IP3R3, IP3R, InsP3R3
Jak2	janus kinase 2	JAK-2, JTK10
Junb	Jun B proto-oncogene	transcription factor jun-B
Kcnh3	potassium voltage-gated channel	
Klf15	Kruppel-like factor 15	
Lmo-4	Lim domain only 4	LIM domain only protein 4, breast tumor autoantigen
Mapk8	mitogen-activated protein kinase 8	Jnk1, SAPK1, JNK ,c-Jun NH2-terminal kinase 1
Mgst1	microsomal glutathione S-transferase 1	
Mrp14	macrophage inhibitor factor-related protein	S100 calcium binding protein A9, CAGB, CFAG
Mt1a	metallothionein 1A	MT-1, MT1S, MT-1A
Mt3	Metallothionein 3	GRIF, MT-3, GIFB, GIF
Nadh2	NADH dehydrogenase subunit 2	
Neurod1	neurogenic differentiation factor	
Ngf1-b	nerve growth factor 1b	ngf1b
Nptx2	neuronal pentraxin II	NP-II, NP2, NARP, apexin
Nqo1	NAD(P)H dehydrogenase, quinone 1	
Nr4a1	nuclear receptor subfamily 4, group A, member 1	HMR, NAK-1, NGFIB, GFRP1, NUR77
N-ras	neuroblastoma RAS viral (v-ras) oncogene homolog	NS6, ALPS4, HRAS
Nrf-1	GA binding protein transcription factor, beta subunit 1	nuclear respiratory factor 2, NRF2B1
Nrf2	nuclear factor-like 2	NFE2L2
Ntm	neurotrimin	HNT, NTRI, hNT
Ntsr2	neurotensin receptor 2	
Nupl1	nucleoporin-like protein 1	nucleoporin p58/p45
Pdia3	protein disulfide isomerase family A, member 3 (calreticulin)	CALR, GRP58, ERp57, ERp60
Pdk2	pyruvate dehydrogenase kinase 2	
PERK	eukaryotic translation initiation factor 2-alpha kinase 3	EIF2AK3, PEK, PRKR-like endoplasmic reticulum kinase
Ppp3	Protein phosphatase 3 (calcineurin)	calcineurin A alpha, CALNA, CNA1, PPP2B, CALN
Ptgs2	prostaglandin-endoperoxide synthase 2	COX-2, Prostaglandin H2 synthase 2, PGHS-2
Ptp4a2	protein tyrosine phosphatase type IVA, member 2	PRL2, HU-PP-1, OV-1
Ptpn1	protein tyrosine phosphatase, non-receptor type 1	PTP-1B
Ret	ret proto-oncogene	HSCR1, cadherin family member 12, PTC, MTC1
Rpl21	ribosomal protein L21	
Scg2	secretogranin II	Chromogranin C, secretogranin 2, CHGC, secretoneurin
Sgk1	serum/glucocorticoid-regulated kinase 1	
Stat3	signal transducer and activator of transcription 3	APRF, HIES, Acute-phase response factor
Sult1a1	aryl sulfotransferase family 1A, phenol-preferring, member 1	St1a1, TSPST1, PST
Syt12	synaptotagmin XII	sytXII, SRG1
Syt4	Synaptotagmin IV	HsT1192, KIAA1342, SytIV
Taf9b	TAF9B RNA polymerase II, TATA binding protein-associated factor, 31kDa	DN-7, neuronal cell death-related protein, Taf9l
Thrb	thyroid hormone receptor, beta	THR1, NR1A2, ERBA-beta
tPA	plasminogen activator, tissue	PLAT, alteplase, reteplase
Trkb	neurotrophic tyrosine kinase, receptor, type 2	GP145-TrkB, trk-B
Ucp2	uncoupling protein 2	UCPH, UCP-2
VAP1	vesicle-associated protein	
Vegfa	vascular endothelial growth factor A	VEGF, VPF, MVCD1
Vgf	nerve growth factor inducible	neurosecretory protein VGF
Vip	vasoactive intestinal peptide	PHM27
Xbp-1	x-box binding protein 1	XBP2, TREB5, XBP-1
